# Case report: A rare case of hereditary hemochromatosis caused by a mutation in the HAMP gene in Fuyang, China

**DOI:** 10.3389/fmed.2024.1417611

**Published:** 2024-06-28

**Authors:** Jinling Wang, Jing Xu, Ning Jiang, Hui Liu, Fengcheng Li, Beibei Wang, Jin Wang, Ziyu Chu, Lin Tan, Shasha Li

**Affiliations:** ^1^Department of Hepatology, The Second People's Hospital of Fuyang City, Fuyang, Anhui, China; ^2^Department of Pathology, Beijing Youan Hospital, Capital Medical University, Beijing, China

**Keywords:** hemochromatosis, siderosis, iron metabolism, gene mutation, bloodletting

## Abstract

Hemochromatosis, also known as siderosis, is a disease caused by excessive iron deposition in human organs and tissues, resulting from iron metabolism disorders. It is clinically characterized by skin pigmentation (bronze color), liver cirrhosis, diabetes, weakness, and fatigue. Additional symptoms may include arthritis, hypothyroidism, heart failure, and sexual hypofunction. Clinical manifestations can vary from person to person, with a few patients showing no clinical manifestations, which makes the diagnosis difficult for clinicians. In this case report, we described hereditary hemochromatosis related to a mutation in the HAMP gene in Fuyang City, China, as a reference for clinicians. Hereditary hemochromatosis is rarely reported in China. Clinicians in China have relatively insufficient knowledge of this disease, which leads to frequent misdiagnosis. In this case report, we describe hereditary hemochromatosis related to HAMP gene mutation in Fuyang City, China, for the clinician’s reference.

## Case information

A 37-year-old male school teacher visited the hepatology department of the Second People’s Hospital of Fuyang City, Anhui Province, on 7 March 2023, with a history of recurrent abnormal liver function for more than 2 years. He was diagnosed with an abnormal liver function during a physical examination in February 2021. Despite undergoing liver protection treatment, the patient’s liver function remained abnormal. There was no more available information related to this case. On 19 February 2023, the patient’s liver function, including alanine aminotransferase (ALT) (126 U/L), and aspartate aminotransferase (AST) (61 U/L), was tested again at another hospital. During his disease progression, the patient was conscious and alert but had a poor diet, weakness, and fatigue, accompanied by occasional pain and discomfort in the finger and toe joints. He reported good sleep and had no obvious abnormalities in urination or defecation. His body weight had not changed significantly over the past 2 years.

### Patient’s clinical history

This patient was married, and had one child. He had no clinical history of diabetes, hypertension, heart disease, infection with viral hepatitis, tuberculosis, surgery, blood transfusions, allergy reports, or other familial diseases. He claimed not to smoke or drink alcohol. The patient’s routine general medical examination data were as follows: conscious and mentally well; a body temperature of 36.6°C; a heart rate of 76 beats per minute (bpm); a respiratory rate of 18 times per minute (tpm); a blood pressure of 125/76 mmHg; and a BMI of 17.6 (underweight). During a physical examination, the patient presented with a soft neck; a flat and soft abdomen without tenderness or rebound pain; and healthy physiological reflexes. The examination ruled out the presence of jaundice of the skin or sclera; obvious abnormalities from heart and lung auscultations; a palpable liver or spleen under the ribs; liver palms and spider nevus; asterixis; moving dullness; and obvious pitting edema in both lower limbs.

### Other laboratory tests

Liver function and blood sugar panels: albumin (44.1 g/L), alanine aminotransferase (53 U/L), aspartate aminotransferase (55 U/L), total bilirubin (13.9 μmol/L), blood sugar (6.17 mmol/L); routine blood tests: white blood cells (3.05*10^ 9/L), red blood cells (4.3*10^12/L), hemoglobin (139 g/L), hematocrit (40.4%), platelets (93*10^9/L); iron metabolism-related indicators: ferritin>1,500 μg/L, serum iron (43 μmol/L), total iron binding capacity (62 μmol/L), unsaturated iron binding capacity (19 μmol/L), and transferrin saturation (69.35%). There were no abnormalities or positive detections in the following clinical characteristics: coagulation function, renal function, blood lipids, cardiac enzymes, urine and feces routine, alpha-fetoprotein, thyroid function, 14 autoantibodies, 12 antinuclear antibodies, immunoglobulins, hepatitis A, B, C, and E, giant cell viruses, and Epstein–Barr virus.

### Imaging examination

**Figure 1:**
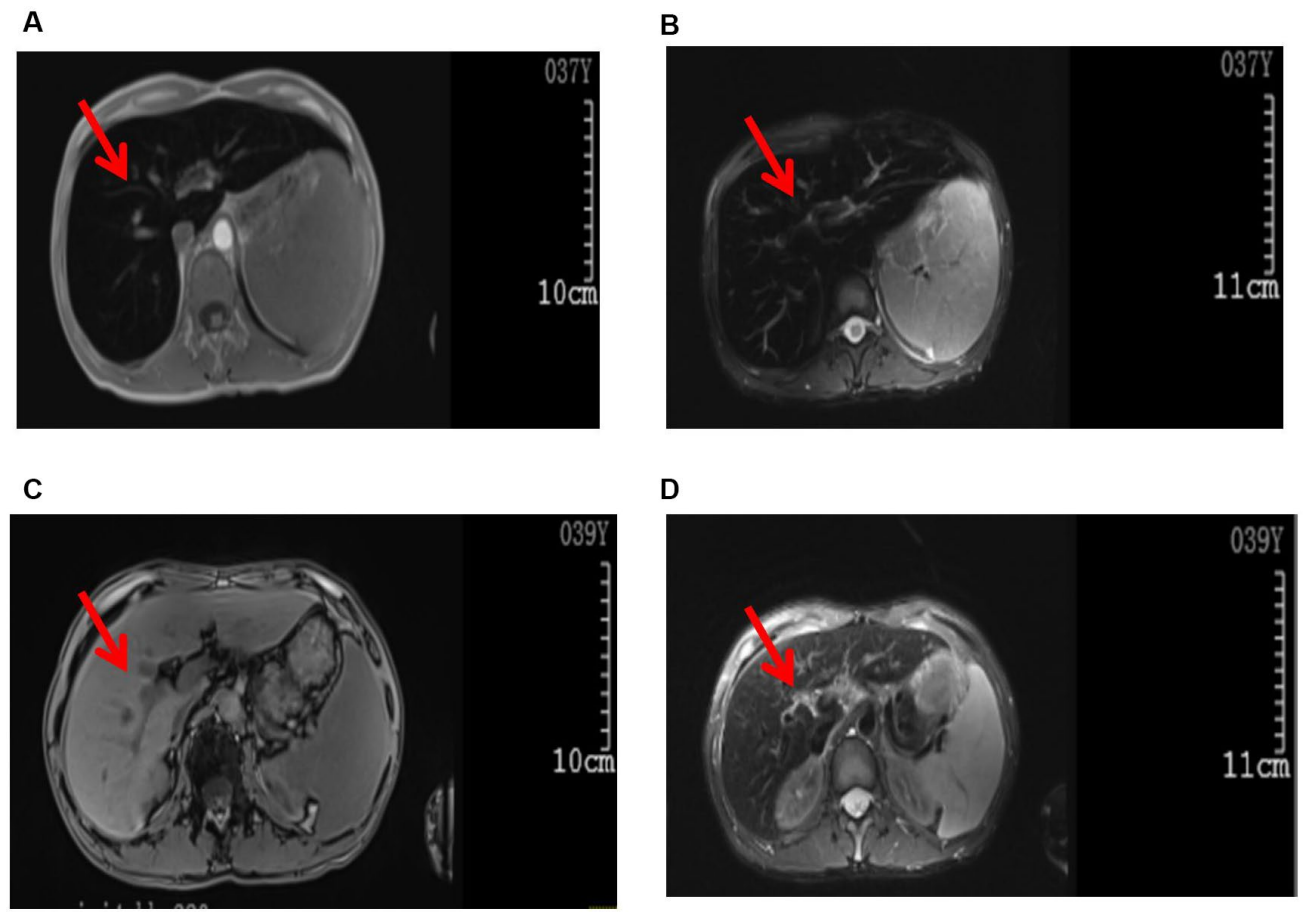
Images of the abdominal MRI. Before treatment (examination time: 9 March 2023): **(A)** T1WI, **(B)** T2W1. The arrows indicate decreased T1W1 and T2W1 signals in the liver parenchyma, presenting as the “black liver syndrome.” After treatment (examination time: 6 May 2024): **(C)** T1WI, **(D)** T2WI. The arrows indicate a significant improvement in T1WI and T2WI signals in the liver parenchyma compared to pre-treatment.

There were no abnormalities detected in the brain parenchyma on the head CT scan, while the plain lung CT scan showed cord shadows, nodular shadows, nodular dense shadows, and bullae in the upper lobe of the left lung. A follow-up was recommended. A color ultrasound of the liver, gallbladder, spleen, and pancreas showed that the liver parenchyma had dense echoes, was slightly thickened, the outline was not clear, and the liver edge was slightly dull. It was considered to be diffuse damage to the liver parenchyma, splenomegaly, ascites (a small amount), hepatic fat infiltration, and splenic vein dilatation. Whole-abdominal CT scan + enhancement showed small nodules on the inner edge of the top of the liver, which were considered to be benign lesions; possible small hemangioma; splenomegaly; accessory spleen; a small cyst in the left kidney; and a small amount of pelvic fluid collection. An MRI plain scan of the liver, the gallbladder, and the spleen (Figures 1A,B) showed no obvious abnormalities in the contour, size, and shape of the liver; the liver capsule was still smooth; the liver fissure was not wide; the shape of the pancreas was normal; the T1WI and T2WI signals of the liver and pancreas were significantly reduced; and the pancreas was inverted. The signal was higher than the signal in the same phase; a small cystic T2WI high signal was observed in the right lobe of the liver with a clear boundary, and no obvious localized abnormal signal was found in the remaining liver parenchyma. The main portal vein was slightly widened with a diameter of approximately 13 mm. There was no obvious dilated in the intrahepatic and intrahepatic bile ducts. The splenic volume was increased, and the T2WI signal was slightly reduced. There was no obvious enlarged lymph node shadow in the retroperitoneal area, a trace signal of effusion around the spleen, and a small amount of effusion in the bilateral pleural cavity. MRI diagnostic conclusions: 1. severe iron deposition in the liver and pancreas and iron deposition in the spleen; 2. portal hypertension, splenomegaly, and trace ascites; 3. small cysts in the liver and left kidney; and 4. a small amount of effusion in the bilateral pleural cavity.

### Pathological examination (liver puncture biopsy)

**Figure 2:**
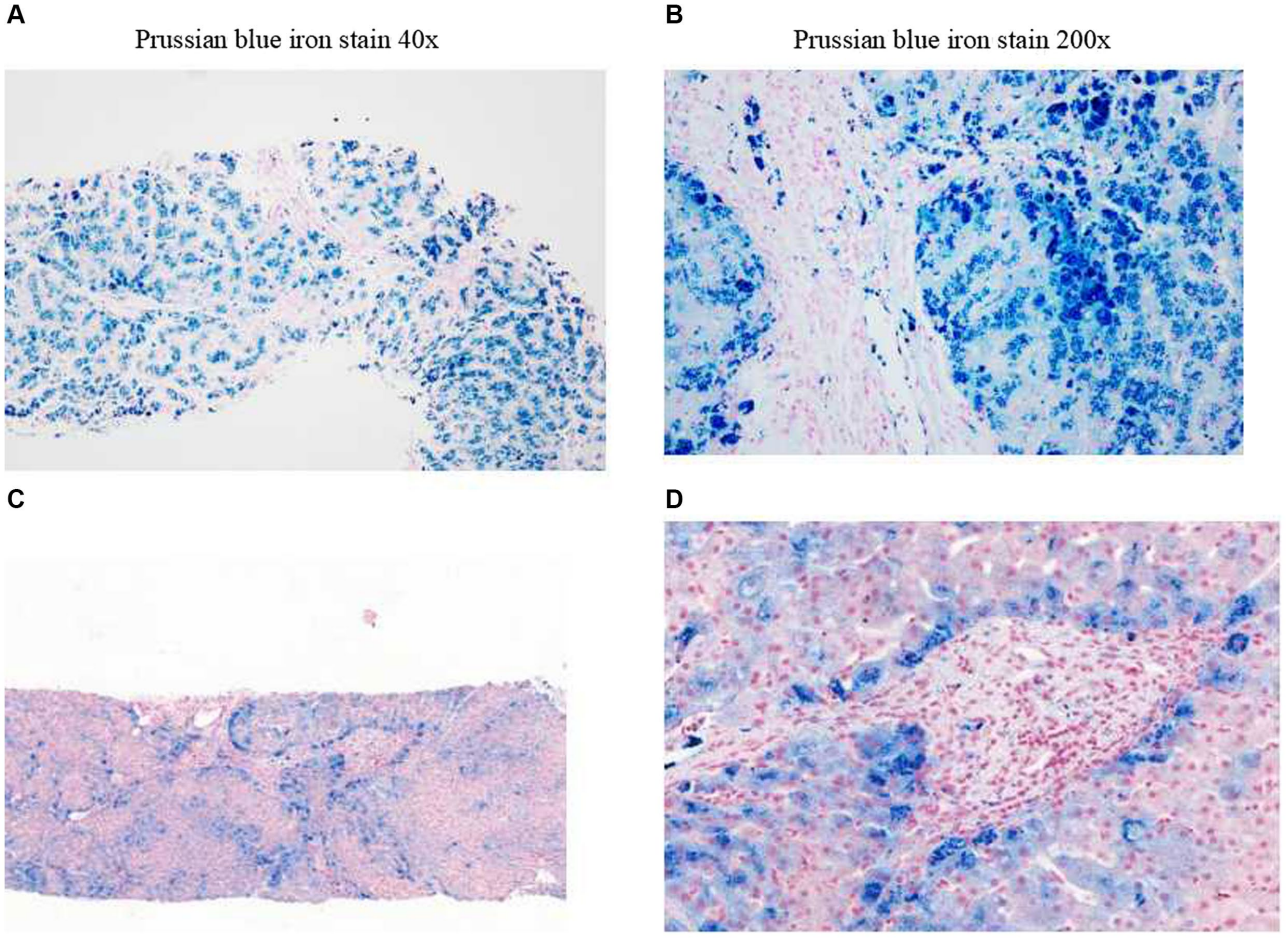
Liver biopsy pathology. Before treatment (examination time: 27 March 2023). **(A)** Prussian blue iron stain, 40x. **(B)** Prussian blue iron stain, 200x; iron overload, Grade 4. After treatment (examination time: 5 May 2024). **(C)** Prussian blue iron stain, 40x. **(D)** Prussian blue iron stain, 200x; iron overload, Grade 2.

The structure of the liver lobules was disordered, there was regional watery degeneration of the liver cells, large coarse particle deposition diffused in the liver cells, iron staining showed iron particle deposition mainly within the liver cells, scattered focal necrosis, a small amount of inflammatory cell infiltration in the liver sinusoids, Kupffer cells engulfing pigment granules, iron particle deposition, the expansion of the portal area, fibrous tissue proliferation, fibrous septa formation, a small amount of inflammatory cell infiltration, and phagocytosis of pigment granules were observed (Figures 2A,B). A clear interface of inflammation was not observed in macrophages (Figures 2A,B). The pathological diagnosis conclusion was as follows: hemosiderosis accompanied by fibrosis formation, with the degree of fibrosis corresponding to S3, copper staining (−), and iron staining (4+) (Figures 2A,B).

### Genetic testing

**Figure 3:**
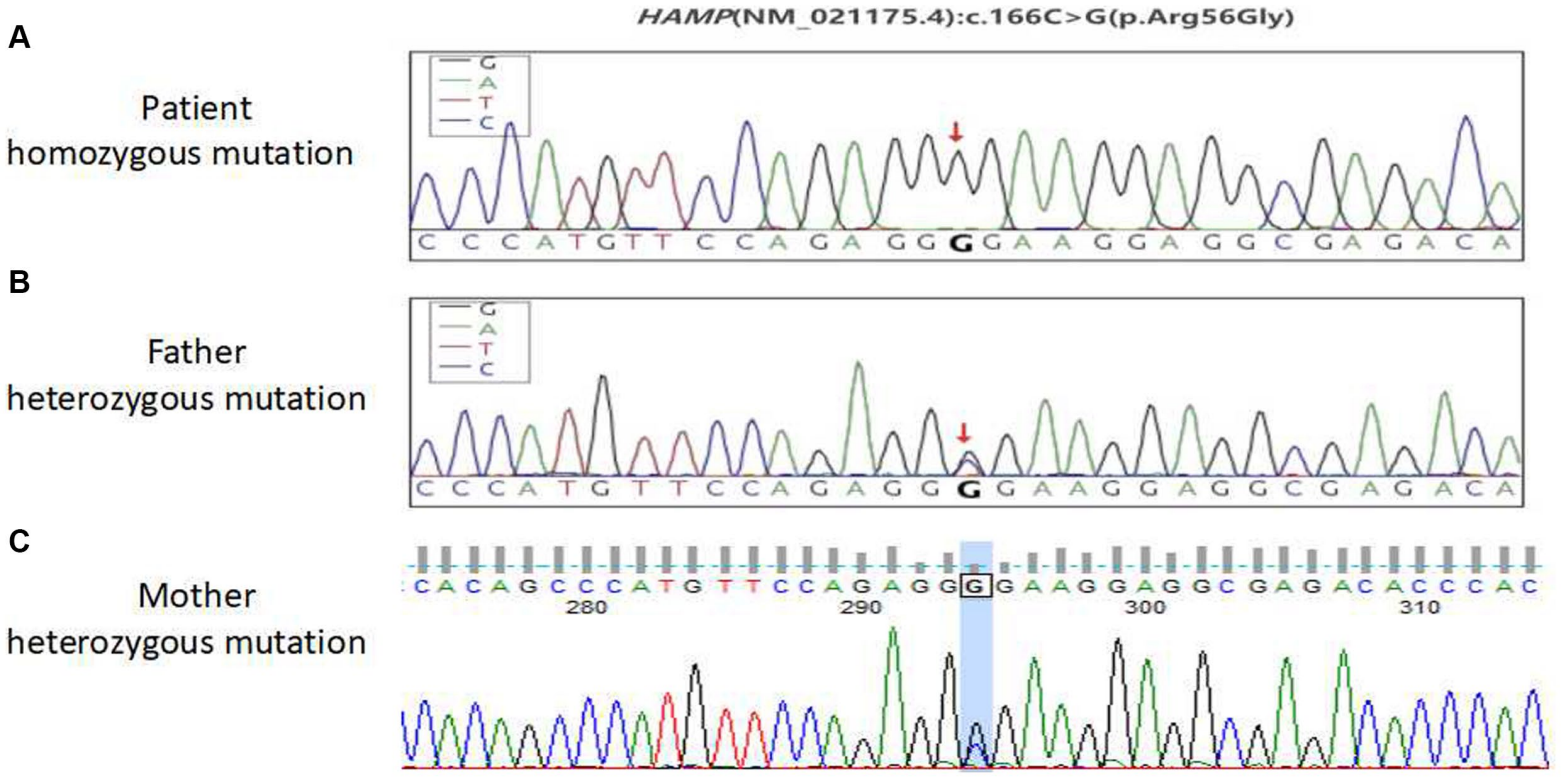
Sequencing peak map of the first pedigree generation with a mutation in the HAMP gene in patients. **(A)** Patient homozygous variants, **(B)** patient paternal heterozygous variants, and **(C)** the patient’s mother is heterozygous.

No abnormal mutations were found in the primary test results. The secondary results detected a variant of unknown clinical significance, the antimicrobial peptide hepcidin (HAMP) variant gene, which may be related to the subject’s clinical information. This was a homozygous HAMP c.166C > G (p.Arg56Gly) mutation. This patient was considered to have primary hemochromatosis due to a mutation in the HAMP gene. To further confirm that the gene mutation was inherited, blood samples from both parents were collected for one-generation verification of the mutation in the HAMP gene. The Sanger sequencing results showed that both patient’s parents had heterozygous mutations at this site (Figure 3). These findings confirmed that the patient’s gene mutation was inherited from his parents. The patient was eventually diagnosed with hereditary hemochromatosis (type 2B).

### Clinical treatment and outcome

In the initial stage of treatment, the patient was prescribed isoglycyrrhizinase for hepatoprotection and enzyme-lowering treatments. He started taking eight tablets of deferasirox daily (125 mg per tablet) orally on 29 March 2023. Due to obvious abdominal distension and discomfort, he discontinued deferasirox in June 2023. The abdominal distension subsided after discontinuing deferasirox. Subsequently, venous bloodletting treatment was started on 29 March 29, 2023. As of 5 May 2024, venous bloodletting treatment has been performed 53 times, with an average of once every week and 400 mL per session, totaling 21,200 mL of blood removal. Since starting this treatment, the patient has not had discomfort symptoms such as chest tightness, palpitations, or dizziness. There has been a noticeable improvement in his symptoms; he reported less weakness and fatigue, improved appetite, and reduced pain and discomfort in his fingers and toe joints. The FE, liver function, and blood sugar levels have gradually returned to the normal range. The iron metabolism, hemoglobin, liver function, and blood sugar indicators of the patient during the treatment are listed in Table 1. MRI showed that the liver parenchyma significantly increased post-treatment (Figures 1C,D). Liver biopsy pathology reported that iron overload was grade 2, and liver fibrosis was grade 2–3; both showed better results than pre-treatment assessments (Figures 2C,D).

**Table 1:** Changes in iron metabolism and liver function indices during treatment.

Characteristics	Reference values	Before Prior TP	TP for 1 M	TP for 3 M	TP for 6 M	TP for 10 M	TP for 14 M
FE(umol/L)	9.0–32.0	43	40.8	41	71.8	36.4	13.9
FER(ug/L)	27.00–375.00	>2000	>1,500	>1,500	>1,500	759.84	75.1
TS(%)	20.0–55.0	69.35	67.1	59.2	29.1	60.2	19.4
Hb(g/L)	130–175	139	128	129	130	130	125
ALT(U/L)	0–50	53	66	29	29	31	15
AST(U/L)	0–40	55	40	48	36	28	16
TBIL(μmol/L)	0–26	13.9	12.1	15.9	14.5	14.1	12.3
IBIL(μmol/L)	0–18	10.8	9.2	13.4	12.2	10.9	8.9
Glu(mmol/L)	3.89–6.11	6.17	5.59	6.02	5.8	5.52	5.57

TP, Therapeutic phlebotomy; M, month; FE, serum iron; FER, ferritin; TS, transferrin saturation; Hb, hemoglobin; ALT, alanine aminotransferase; AST, aspartate aminotransferase; Glu, blood glucose.

## Discussion

Hereditary hemochromatosis is an autosomal recessive disorder of iron metabolism characterized by increased intestinal iron absorption and iron release by macrophages. This results in an expansion of circulating iron pools, reflected by the increase in transferrin saturation and leads to a progressive accumulation of iron in the body, mainly deposited in the liver (1). If left untreated, it can lead to liver fibrosis, cirrhosis, and even hepatocellular carcinoma (HCC) (2, 3). Hemochromatosis has a wide range of clinical manifestations (4–6). In addition to abnormal liver enzymes and liver fibrosis, it can also show elevated blood sugar, hyposexuality, bronze skin, arthritis, joint pain, arrhythmia, heart failure, etc., which causes great challenges to a patient’s health. Men are significantly more affected than women, and the incidence also increases with age (7). Hereditary hemochromatosis could be easily ignored in terms of diagnoses in China due to the rarity of this condition (4, 5). This patient’s clinical presentation included repeated abnormal liver function as the first symptom and clinical manifestations of fatigue, mild pain in the joints of the fingers and toes, elevated blood sugar, and liver fibrosis. All of these clinical characteristics were consistent with the typical clinical manifestations of hereditary hemochromatosis. Hemochromatosis was suspected in the initial diagnosis because the abdominal MRI scan showed severe iron deposition in the liver and the spleen. The diagnosis of type 2B hereditary hemochromatosis in this patient was finally confirmed through a liver biopsy and genetic testing.

In laboratory test results, hemochromatosis was characterized by markedly elevated transferrin saturation and serum ferritin. The color Doppler ultrasound and CT examination of the liver of the patient showed no specific findings, and the liver MRI examination showed a significant reduction in liver parenchymal signal (8). According to the recommendations of the nomenclature committee of the International Society for Iron Research in Biology and Medicine (9), hereditary hemochromatosis can be classified into four categories: homeostatic iron regulator (HFE-related) (p. Cys282Tyr homozygous gene mutation, p.Cys282Tyr/His63 Asp heterozygous gene mutation); non-HFE-related, including hemojuvelin (HJV), HAMP, transferrin receptor 2(TFR2), solute carrier family 40 member 1 (SLC40A1) related gene mutations; mixed (mixed mutations of HFE-related and non-HFE-related genes); or not yet classified (provisional diagnosis: after sequencing known genes, molecular characteristics are temporarily unavailable). Hereditary hemochromatosis is more common in European populations, with a prevalence ranging from 1:83 in Ireland to 1:2500 in southern Europe (10). In patients of European origin, homozygous HFEp.Cys282Tyr mutations have been found in 80% of patients (11). The patient in this report has type 2B hereditary hemochromatosis caused by a rare HAMP gene mutation, which is also known as juvenile hereditary hemochromatosis (12). Compared with hereditary hemochromatosis caused by HFE and TFR2, HAMP gene mutation has a greater risk of heart attack, skin changes, liver fibrosis, and hypogonadism (13). It has been demonstrated that HAMP gene knockout mice can develop intrahepatic iron overload at 2 months of age, and with increasing age, iron deposition both inside and outside of the liver increases rapidly (14). Hereditary hemochromatosis with mutations in the HAMP gene can lead to islet autoantibody-positive type 1 diabetes (15). This patient had high blood sugar before treatment. The first line of treatment for hemochromatosis is phlebotomy, which is performed to reduce iron accumulation in the body (16). Venous bloodletting treatment can improve fatigue, joint pain, and liver function indicators, reducing liver fibrosis and cirrhosis in some patients (1). The morbidity and mortality in patients with hemochromatosis are significantly reduced when phlebotomy treatment is initiated before the development of cirrhosis and/or diabetes (17). Following venous bloodletting treatment in this patient, we observed that serum ferritin and serum iron levels were significantly reduced, fatigue and joint pain were alleviated, blood sugar returned to the normal range, and liver function and liver fibrosis improved. We have not found further adverse reactions since the venous bloodletting treatment. The limitation of this report is that it is still an ongoing treatment case. It is unclear whether the patient’s liver function and serum iron level will stabilize after stopping the venous bloodletting therapy.

In summary, the incidence of hereditary hemochromatosis related to mutations in the HAMP gene is rare in China. Insidious onset and diverse clinical manifestations in patients may lead to misdiagnosis by clinicians. This patient’s hereditary hemochromatosis was identified due to the typical “black liver disease” presented in the liver MRI. The identification and treatment process in this report may provide valuable insights and ideas to front-line clinicians in the diagnosis and therapy of hemochromatosis.

## Data availability statement

The original contributions presented in the study are included in the article/supplementary material, further inquiries can be directed to the corresponding authors.

## Ethics statement

The studies involving humans were approved by Ethics Committee of the Second People's Hospital of Fuyang City. The studies were conducted in accordance with the local legislation and institutional requirements. The participants provided their written informed consent to participate in this study. Written informed consent was obtained from the individual(s) for the publication of any potentially identifiable images or data included in this article.

## Author contributions

JinlW: Writing – original draft, Methodology, Investigation, Formal analysis, Data curation. JX: Writing – original draft, Methodology, Investigation, Formal analysis. NJ: Writing – original draft, Methodology, Investigation. HL: Writing – original draft, Writing – review & editing. FL: Writing – original draft, Investigation. BW: Writing – original draft, Investigation. JinW: Writing – original draft, Investigation. ZC: Writing – original draft, Investigation. LT: Writing – review & editing, Investigation. SL: Writing – review & editing, Writing – original draft.

## References

[1] European Association for the Study of the Liver. Electronic address eee, European Association for the Study of the L. Easl clinical practice guidelines on haemochromatosis. J Hepatol. (2022) 77:479–502. doi: 10.1016/j.jhep.2022.03.033, PMID: 35662478

[2] Atkins JL Pilling LC Masoli JAH Kuo CL Shearman JD Adams PC. Association of Hemochromatosis Hfe P.C282y homozygosity with hepatic malignancy. JAMA. (2020) 324:2048–57. doi: 10.1001/jama.2020.21566, PMID: 33231665 PMC7686863

[3] Pilling LC Tamosauskaite J Jones G Wood AR Jones L Kuo CL. Common conditions associated with hereditary haemochromatosis genetic variants: cohort study in Uk biobank. BMJ. (2019) 364:k5222. doi: 10.1136/bmj.k5222, PMID: 30651232 PMC6334179

[4] Tang S Bai L Gao Y Hou W Song W Liu H. A novel mutation of transferrin receptor 2 in a Chinese pedigree with type 3 hemochromatosis: a case report. Front Genet. (2022) 13:836431. doi: 10.3389/fgene.2022.836431, PMID: 35464850 PMC9024051

[5] Zhang W Wang X Duan W Xu A Zhao X Huang J. Hfe-related hemochromatosis in a Chinese patient: the first reported case. Front Genet. (2020) 11:77. doi: 10.3389/fgene.2020.00077, PMID: 32153640 PMC7048005

[6] Kowdley KV Brown KE Ahn J Sundaram V. Acg clinical guideline: hereditary hemochromatosis. Am J Gastroenterol. (2019) 114:1202–18. doi: 10.14309/ajg.0000000000000315, PMID: 31335359

[7] Hagstrom H Ndegwa N Jalmeus M Ekstedt M Posserud I Rorsman F. Morbidity, risk of Cancer and mortality in 3645 Hfe mutations carriers. Liver Int. (2021) 41:545–53. doi: 10.1111/liv.14792 33450138

[8] Guyader D Gandon Y Robert JY Heautot JF Jouanolle H Jacquelinet C. Magnetic resonance imaging and assessment of liver Iron content in genetic hemochromatosis. J Hepatol. (1992) 15:304–8. doi: 10.1016/0168-8278(92)90060-3, PMID: 1447496

[9] Girelli D Busti F Brissot P Cabantchik I Muckenthaler MU Porto G. Hemochromatosis classification: update and recommendations by the bioiron society. Blood. (2022) 139:3018–29. doi: 10.1182/blood.2021011338, PMID: 34601591 PMC11022970

[10] European Association for the Study of the Liver. European association for the study of the L. Easl clinical practice guidelines for Hfe hemochromatosis. J Hepatol. (2010) 53:3–22. doi: 10.1016/j.jhep.2010.03.001, PMID: 20471131

[11] Porto G Brissot P Swinkels DW Zoller H Kamarainen O Patton S. Emqn best practice guidelines for the molecular genetic diagnosis of hereditary hemochromatosis (Hh). Eur J Hum Genet. (2016) 24:479–95. doi: 10.1038/ejhg.2015.128, PMID: 26153218 PMC4929861

[12] Papanikolaou G Samuels ME Ludwig EH MacDonald ML Franchini PL Dube MP. Mutations in Hfe2 cause Iron overload in chromosome 1q-linked juvenile hemochromatosis. Nat Genet. (2004) 36:77–82. doi: 10.1038/ng1274, PMID: 14647275

[13] Sandhu K Flintoff K Chatfield MD Dixon JL Ramm LE Ramm GA. Phenotypic analysis of hemochromatosis subtypes reveals variations in severity of Iron overload and clinical disease. Blood. (2018) 132:101–10. doi: 10.1182/blood-2018-02-830562, PMID: 29743178

[14] Zumerle S Mathieu JR Delga S Heinis M Viatte L Vaulont S. Targeted disruption of Hepcidin in the liver recapitulates the Hemochromatotic phenotype. Blood. (2014) 123:3646–50. doi: 10.1182/blood-2014-01-550467, PMID: 24646470

[15] Wu HX Liu JY Yan DW Li L Zhuang XH Li HY. Atypical juvenile hereditary hemochromatosis onset with positive pancreatic islet autoantibodies diabetes caused by novel mutations in Hamp and Overall clinical management. Mol Genet Genomic Med. (2020) 8:e1522. doi: 10.1002/mgg3.1522, PMID: 33016646 PMC7767552

[16] Brissot P de Bels F. Current approaches to the Management of Hemochromatosis. Hematology Am Soc Hematol Educ Program. (2006) 2006:36–41. doi: 10.1182/asheducation-2006.1.36 17124037

[17] Prabhu A Cargill T Roberts N Ryan JD. Systematic review of the clinical outcomes of Iron reduction in hereditary hemochromatosis. Hepatology. (2020) 72:1469–82. doi: 10.1002/hep.31405, PMID: 32500577

